# Hepatitis B virus surface proteins accelerate cholestatic injury and tumor progression in Abcb4-knockout mice

**DOI:** 10.18632/oncotarget.15003

**Published:** 2017-02-02

**Authors:** Daniel Zahner, Hannah Glimm, Tomomitsu Matono, Yuri Churin, Diran Herebian, Ertan Mayatepek, Kernt Köhler, Stefan Gattenlöhner, Anne Stinn, Annette Tschuschner, Martin Roderfeld, Elke Roeb

**Affiliations:** ^1^ Central Laboratory Animal Facility, Justus Liebig University, Giessen, Germany; ^2^ Department of Gastroenterology, Justus Liebig University, Giessen, Germany; ^3^ Department of General Pediatrics, Neonatology and Pediatric Cardiology, Medical Faculty, Heinrich-Heine-University, Duesseldorf, Germany; ^4^ Institute of Veterinary Pathology, Justus-Liebig-University, Giessen, Germany; ^5^ Department of Pathology, Justus-Liebig-University, Giessen, Germany

**Keywords:** HBsAg, ER-stress, carcinogenesis, cholangitis, fibrosis

## Abstract

Understanding of the pathophysiology of cholestasis associated carcinogenesis could challenge the development of new personalized therapeutic approaches and thus improve prognosis. Simultaneous damage might aggravate hepatic injury, induce chronic liver disease and even promote carcinogenesis. We aimed to study the effect of Hepatitis B virus surface protein (HBsAg) on cholestatic liver disease and associated carcinogenesis in a mouse model combining both impairments. Hybrids of Abcb4^−/−^ and HBsAg transgenic mice were bred on fibrosis susceptible background BALB/c. Liver injury, serum bile acid concentration, hepatic fibrosis, and carcinogenesis were enhanced by the combination of simultaneous damage in line with activation of c-Jun N-terminal kinase (JNK), proto-oncogene c-Jun, and Signal transducer and activator of transcription 3 (STAT3). Activation of Protein Kinase RNA-like Endoplasmic Reticulum Kinase (PERK) and Eukaryotic translation initiation factor 2A (eIF2α) indicated unfolded protein response (UPR) in HBsAg-expressing mice and even in Abcb4^−/−^ without HBsAg-expression. CONCLUSION: Cholestasis-induced STAT3- and JNK-pathways may predispose HBsAg-associated tumorigenesis. Since STAT3- and JNK-activation are well characterized critical regulators for tumor promotion, the potentiation of their activation in hybrids suggests an additive mechanism enhancing tumor incidence.

## INTRODUCTION

Various phenotypes of chronic liver disease like progressive familial intrahepatic cholestasis (PFIC type 3) or biliary liver cirrhosis have been attributed to mutations of the human *ABCB4* gene [1]. The *ABCB4* gene encodes the P-glycoprotein ABCB4, a member of the ATP-binding cassette (ABC) transporters, which is responsible for phospholipid transport across the canalicular membrane. ABCB4 harbors numerous missense mutations which probably reflect the spectrum of liver disease depending on ABCB4 background [2]. The lack of Abcb4 transporters in mice causes a complete absence of micelle forming phospholipids from bile, which results in liver injury from chronic cholangitis [3]. Abcb4 knockout mice (Abcb4^−/−^) represent a highly reproducible, well characterized non-surgical mouse model for cholangiopathy [3].

Hepatic injury in patients with chronic hepatitis B virus (HBV) infection has mainly been attributed to T cell response against infected hepatocytes [4, 5]. Nevertheless, observations in HBV infected patients under conditions of immune suppression and in transgenic mouse models of HBV infection suggest, that cellular modifications induced by HBsAg proteins may also lead to liver disease in the absence of adaptive immune responses [6–8]. In HBV infected humanized uPA-SCID chimeric mice the majority of human hepatocytes had ground-glass appearance, stained intensely for viral proteins, and showed considerable hepatic damage and cell death [6]. Interestingly, transgenic mice expressing HBV surface proteins demonstrated comparable histopathologic pattern including inflammation, regenerative hyperplasia, transcriptional deregulation, aneuploidy progressing to neoplasia, and even hepatocellular carcinoma (HCC) [6, 9–11]. Underlying pathomechanisms are based on hepatic accumulation of HBsAg inducing ER stress and subsequent activation of UPR [10–12]. HBsAg transgenic mice (HBsAg^+/−^) are largely tolerant towards transgenic proteins [13] and thus represent an excellent model for the investigation of direct cytotoxic effects of HBsAg and protein storage disease [11, 14, 15]. A higher HBsAg level can predict disease progression [16]. HBsAg is an important predictor of hepatocellular carcinoma (HCC) in patients with chronic HBV infection as hepatocarcinogenesis is predominantly observed in patients with high HBsAg levels [17]. Furthermore, Trierweiler et al. demonstrated that the transcription factor c-JUN/AP-1 promotes HCC in HBsAg-transgenic mice [18].

Additionally, it has been suggested that the accumulation of a misfolded protein in the liver can sensitize to other injuries [19]. The aim of this study was to characterize aggravation of cholestatic liver disease and carcinogenesis by HBsAg expression in Abcb4^−/−^-mice. We hypothesized that hepatic expression of HBsAg in mice lacking the phospholipid flippase multidrug resistance protein 2 (Abcb4^−/−^) represents a suitable model to analyze simultaneous damage by direct cytopathic effects of HBsAg and chronic biliary liver disease. In this study, we established a murine HBsAg^+/−^/Abcb4^−/−^- model and characterized mechanisms leading to enhanced liver injury and carcinogenesis.

## RESULTS

### Elevated liver injury in Abcb4^−/−^/HBsAg^+/−^ mice

**Figure 1:**
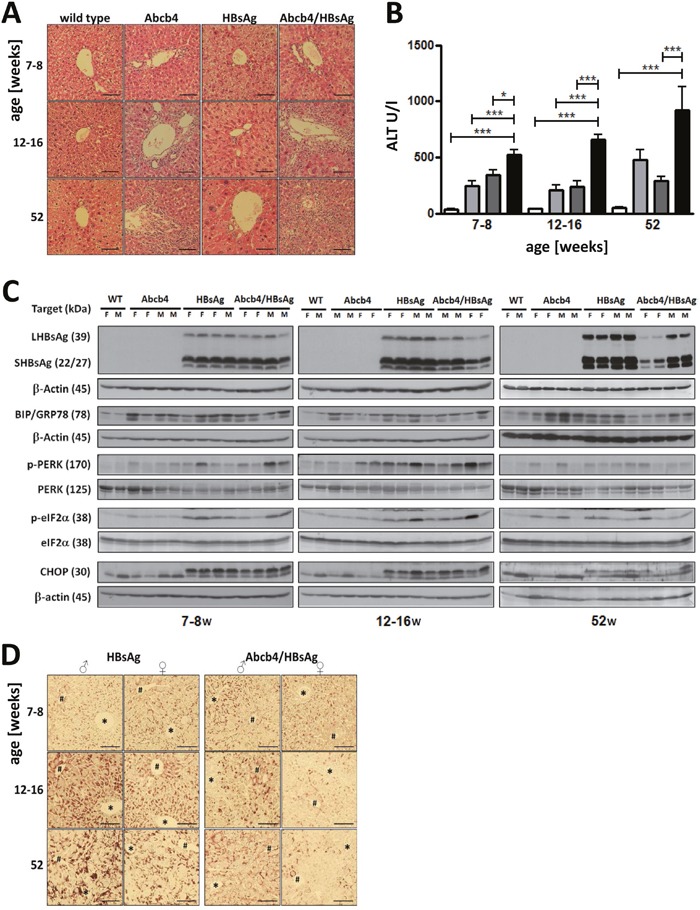
HBsAg expression elevates liver injury in Abcb4 knockout mice **A.** Liver histology demonstrates accelerated portal inflammation and pronounced bile duct disease in Abcb4^−/−^/HBsAg^+/−^ mice. Magnification x200, scale bars 100 μm. **B.** HBsAg expression increases serum ALT in Abcb4^−/−^ mice. Open bars represent wild types, light grey bars Abcb4^−/−^, dark grey bars HBsAg^+/−^, and black bars Abcb4^−/−^/HBsAg^+/−^. **C.** Western blotting demonstrates reduced expression of HBsAg and ER stress in Abcb4^−/−^/HBsAg^+/−^ mice. **D.** Immunohistochemistry visualizes reduced HBsAg expression in female Abcb4^−/−^/HBsAg^+/−^ mice. Representative micrographs are shown.

Abcb4^−/−^/HBsAg^+/−^ mice demonstrated age related increasing infiltration of immune cells in portal fields compared with controls (Figure 1A). Significant destruction of liver architecture and pronounced localized compensatory proliferation were most prominent in the liver of Abcb4^−/−^/HBsAg^+/−^ animals. Proliferation of bile ducts in Abcb4^−/−^ mice was enforced by HBsAg expression in Abcb4^−/−^/HBsAg^+/−^ mice. Serum ALT was significantly increased in all transgenic mice. Levels increased 2-3-fold in Abcb4^−/−^/HBsAg^+/−^ animals in comparison to Abcb4^−/−^ controls (Figure 1B). Moreover, ALT in Abcb4^−/−^/HBsAg^+/−^ mice nearly reflects the sum of ALT levels in Abcb4^−/−^ and HBsAg^+/−^ controls. During the observation period, serum ALT levels raised from 521 U/L at the age of 7-8 weeks up to 920 U/L in 52 weeks old Abcb4^−/−^/HBsAg^+/−^ animals.

### Reduced HBsAg expression in Abcb4^−/−^/HBsAg^+/−^ mice did not influence UPR

In order to compare hepatic transgene accumulation in HBsAg^+/−^- and Abcb4^−/−^/HBsAg^+/−^ mice, HBsAg was analyzed by Western blotting and immunohistochemistry (Figures 1C and 1D). Western blotting revealed expression of large (LHBsAg) and small (SHBsAg) HBsAg. Neither LHBsAg nor SHBsAg expression was found in wild type and Abcb4^−/−^ mice. HBsAg expression was reduced in Abcb4^−/−^/HBsAg^+/−^ mice compared to HBsAg^+/−^ controls at ages of 12-16 weeks and 52 weeks. Hepatic accumulation of HBsAg was stronger in male than in female transgenic mice. A reduction of transgene expression was observed in female and male Abcb4^−/−^/HBsAg^+/−^ mice in comparison to HBsAg^+/−^ controls (Figure 1C and 1D). Immunostaining demonstrated HBsAg accumulation in all hepatocytes of Abcb4^−/−^/HBsAg^+/−^ mice. The amount of intracellular HBsAg, however, was reduced in comparison to HBsAg^+/−^ control mice (Figure 1D) negative controls for the staining of HBsAg in wild type mice and Abcb4^−/−^ mice shown in Supplementary Figure 1. In order to investigate the effect of cholestasis on HBsAg-associated ER stress [11, 12], activation of GRP78, PERK, eIF2a, and Chop was analyzed by Western blotting. Phosphorylation of PERK, eIF2α, and ER stress induced GRP78 and CHOP expression were similar in Abcb4^−/−^/HBsAg^+/−^ mice compared with HBsAg^+/−^ mice (Figure 1C). Interestingly, GRP78, PERK and eIF2α were also activated in Abcb4^−/−^ mice. This effect was most prominent in 52 weeks old mice. Nevertheless, expression of CHOP was missing in Abcb4^−/−^ controls.

### Serum bile acid concentrations were potentiated in Abcb4^−/−^/HBsAg^+/−^ mice

**Figure 2:**
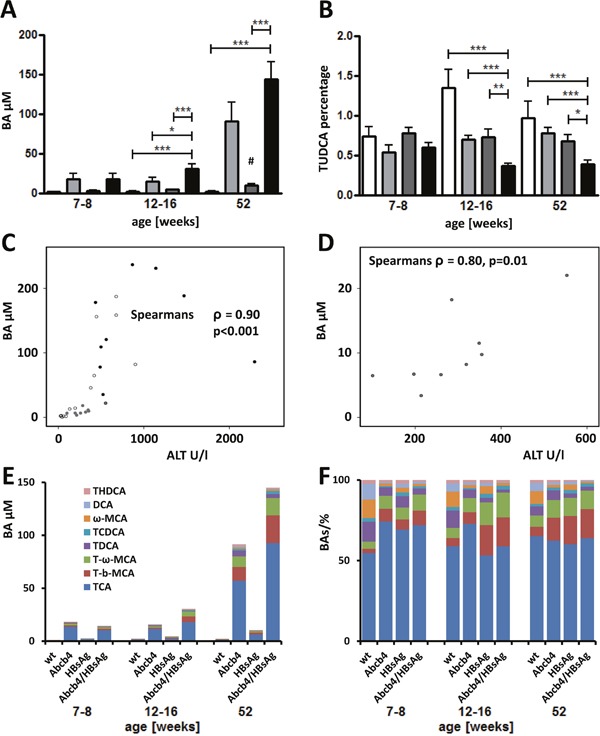
Total serum BA levels raise while relative amount of protective BAs decrease in Abcb4^−/−^/HBsAg^+/−^ mice **A.** Total serum taurine conjugated Bas (T-BAs) increased in Abcb4^−/−^/HBsAg^+/−^-mice in comparison to controls. Serum T-BAs were measured by UHPLC-MS/MS at the day of sacrifice. Total T-BAs are depicted. Note, total T-BAs were increased in Abcb4^−/−^-mice in comparison to wt controls and in HBsAg^+/−^ transgenic mice aged 52 weeks as well (#). **B.** Relative amount of protective TUDCA was decreased in Abcb4^−/−^/HBsAg^+/−^-mice. Results are presented as means ± SEM, n=4-10. *p<0.05 as indicated, ^#^p=0.001 in comparison to wt control. Open bars represent wild types, light grey bars Abcb4^−/−^, dark bars HBsAg^+/−^, and black bars Abcb4^−/−^/HBsAg^+/−^. **C.** Scatter-plot demonstrates the correlation of ALT and total serum T-BA levels. All data of 52 week old animals are included; open dots wt, grey dots Abcb4^−/−^, dark grey dots HBsAg^+/−^, black dots Abcb4^−/−^/HBsAg^+/−^. **D.** Scatter-plot demonstrates the correlation of ALT and total serum T-BA levels in HBsAg^+/−^-mice. **E** and **F.** Absolute- (E) and relative (F) composition of the 8 most abundant bile acids in serum.

Serum concentrations of 11 analyzed Taurine conjugates were combined to illustrate the global increase in serum bile acid concentrations in Abcb4^−/−^/HBsAg^+/−^ (Figure 2A). Bile acid concentrations were also enhanced in HBsAg transgenic mice in comparison to wild type controls aged 52 weeks (^#^p=0.001). Unconjugated bile acids and glycine conjugates were detected at negligible levels and thus were excluded from further analysis. Interestingly, relative serum levels of TUDCA, a protective bile acid, were significantly reduced in Abcb4^−/−^/HBsAg^+/−^ mice (Figure 2B). Serum ALT and bile acid concentrations correlated well, even in HBsAg^+/−^ controls (Figure 2C and 2D). Figure 2E and 2F demonstrate the absolute- and relative composition of the 8 most abundant bile acids in serum. Complete data of all bile acids are presented in Supplementary Table 1.

### HBsAg expression increased fibrosis and biliary proliferation in Abcb4^−/−^/HBsAg^+/−^ mice

**Figure 3:**
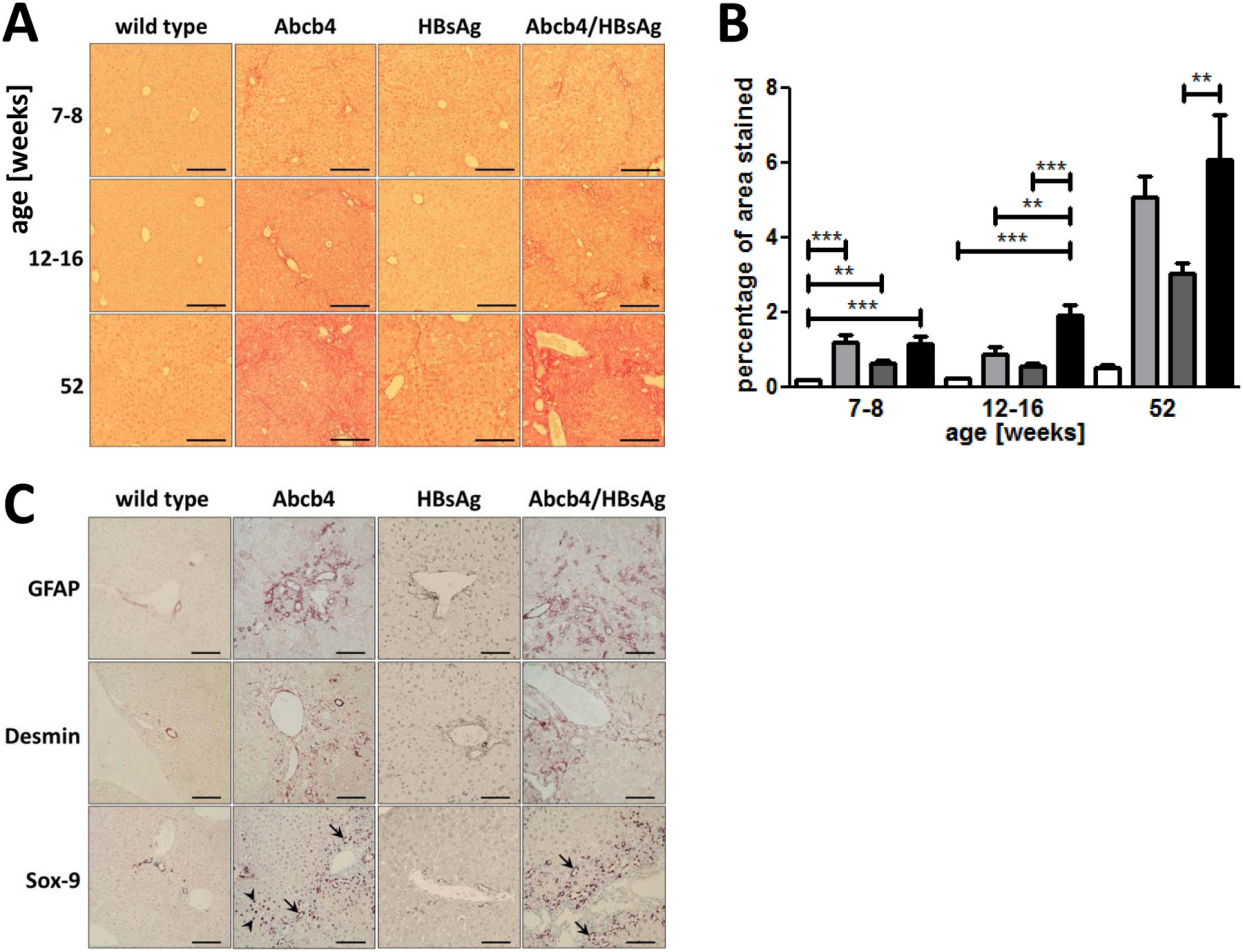
Hepatic fibrosis is elevated in Abcb4^−/−^/HBsAg^+/−^ mice **A.** Representative Sirius red staining demonstrates enhanced collagen accumulation in Abcb4^−/−^/HBsAg^+/−^ mice. Magnification x100, scale bars 200 μm. **B.** Quantification of SiriusRed positive area was performed by computer-analysis (ImageJ). Open bars represent wild types, light grey bars Abcb4^−/−^, dark grey bars HBsAg^+/−^, and black bars Abcb4^−/−^/HBsAg^+/−^. **C.** HBsAg expression enhanced HSC activation and ductular reaction. Representative immunohistochemical stainings for quiescent HSC marker GFAP, HSC activation marker desmin, and transcription factor SOX9 in 12-16 week old mice. Magnification x100, scale bars 100μm. Results are presented as means ± SEM, n=6-10. *p<0.05 as indicated.

Fibrotic fibers were visualized by Sirius red (SR) staining (Figure 3A). Onion-skin-like periductular fibrosis already developed in young Abcb4^−/−^ and Abcb4^−/−^/HBsAg^+/−^ mice. The gain of periportal and septal fibrosis characterized progressive fibrosis in Abcb4^−/−^/HBsAg^+/−^ mice. Quantification of the SR-stained area of the tissue revealed significant fibrosis in liver injury models at all ages (Figure 3B). Abcb4^−/−^/HBsAg^+/−^ mice aged 12-16 week showed enhanced fibrosis compared to controls (wild type: 0.24±0.09%, Abcb4^−/−^ 0.90±0.58%, HBsAg^+/−^ 0.57±0.16%, and Abcb4^−/−^/HBsAg^+/−^ 1.19±0.58%). Glial fibrillary acidic protein (GFAP)- and desmin positive cells appeared more frequently in Abcb4^−/−^/HBsAg^−/−^ mice, especially around portal tracts (Figure 3C), indicating enhanced activation of hepatic stellate cells. Immunostaining for SOX9 (biliary marker) indicated ductular reaction in Abcb4^−/−^ mice (arrows Figure 3C). Furthermore, SOX9^+^ cells of hepatocyte shape indicated the participation of chronically injured hepatocytes within ductular reactions in Abcb4^−/−^- and Abcb4^−/−^/HBsAg^+/−^ mice (arrowheads Figure 3C) [20, 21].

### Abcb4^−/−^/HBsAg^+/−^ mice display enhanced carcinogenesis

**Figure 4:**
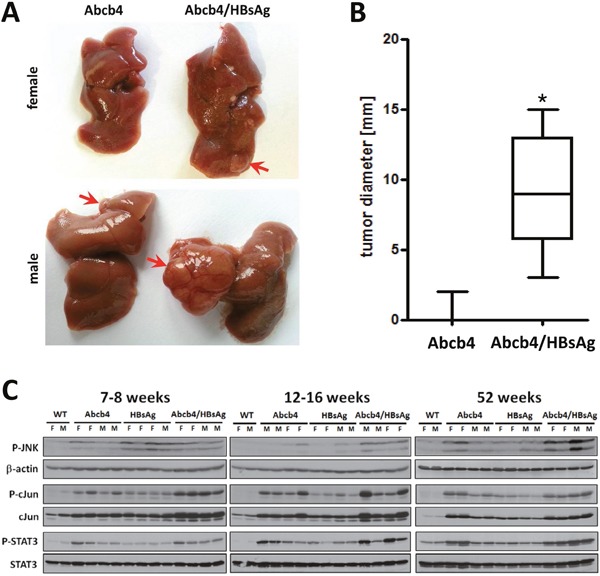
Induction of carcinogenic pathways enhanced tumor incidence in Abcb4^−/−^/HBsAg^+/−^ mice **A.** Macroscopic view of well vascularized primary tumors > 5mm in diameter which appeared more frequently in Abcb4^−/−^/HBsAg^+/−^ mice. **B.** Tumor diameter is shown in Box and Whisker Plots. Enhanced incidence was calculated with Chi^2^ statistics. The upper and lower hinges of the box represent the 75th and 25th percentile, respectively. The line indicates the median value; error bars represent the minimum and maximum. **C.** Western blot analysis of phospho-SAPK/JNK (P-JNK), β-actin, phospho-c-Jun (P-cJun), c-Jun, phospho-STAT3 (P-STAT3), and STAT3 demonstrate enhanced induction of carcinogenic pathways in Abcb4^−/−^/HBsAg^+/−^ mice.

Hepatic tumors were observed in 8 out of 9 Abcb4^−/−^/HBsAg^+/−^ mice aged 52 weeks (Ø=8.3±4.5 mm) whereas in Abcb4^−/−^ controls only 1 out of 9 animals (Ø=2mm) developed tumors (Figure 4A and 4B). Hence, development of hepatic tumors in Abcb4^−/−^/HBsAg^+/−^ animals was significantly increased by HBsAg expression in comparison to Abcb4^−/−^ controls (X^2^=10.9, p=0.001). Increased tumor size went along with pronounced angiogenesis in Abcb4^−/−^/HBsAg^+/−^ mice (Figure 4A, lower right panel). We recently described tumorigenesis in HBsAg^+/−^ controls (58% of male and 0% of female HBsAg^+/−^ mice aged 52 weeks) [11]. Neither wild type mice nor young mice developed any tumors at all.

In order to characterize signaling pathways promoting hepatic tumor growth, phosphorylation of c-Jun, c-Jun N-terminal kinase (JNK), as well as STAT-3 were analyzed by Western blot analysis of hepatic protein extracts. c-Jun expression as well as c-Jun phosphorylation were enhanced in Abcb4^−/−^/HBsAg^+/−^ mice suggesting cumulative effects of both murine models (Figure 4C). In addition, activation of JNK was enhanced in Abcb4^−/−^/HBsAg^+/−^ mice compared to controls. STAT3 activation was stronger in Abcb4^−/−^ mice in comparison to HBsAg^+/−^ controls and appeared with nearly additive signal intensities in Abcb4^−/−^/HBsAg^+/−^ mice.

**Figure 5:**
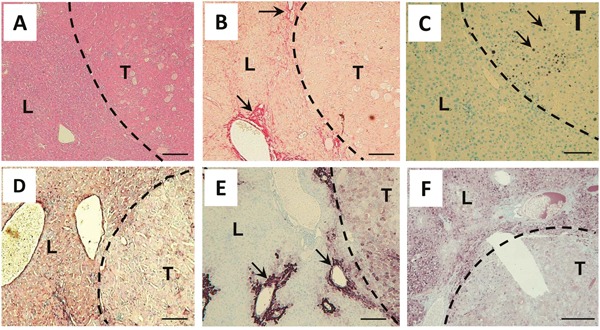
Tumors in Abcb4^−/−^/HBsAg^+/−^ mice show histological characteristics of HCC **A.** A representative H&E staining demonstrates well defined tumor borders (dashed line). **B.** Tumor stroma is nearly free from Sirius red staining fibrillar collagen. Arrows point out fibrotic area in surrounding liver tissue. **C.** Ki67 staining (arrows) demonstrates pronounced proliferation in tumor stroma. **D.** Type IV collagen immunostaining depicts a lack of expression inside tumor stroma. **E.** Immunostaining for glutamine synthetase depicts expression in pericentral hepatocytes (arrows) and diffuse expression pattern in tumor stroma. **F.** Immunostaining for HBsAg demonstrates the loss of transgene expression in tumors. L liver, T tumor. Magnification x100 A-F, scale bars 100μm.

Histologically, tumors were characterized by a defined tumor border (Figure 5A), lack of fibrillary collagen (Figure 5B), remarkable cellular proliferation (Figure 5C), lack of type IV collagen in the tumor parenchyma (Figure 5D), diffuse glutamine synthetase expression pattern (Figure 5E), and a lack of HBsAg expression (Figure 5F).

## DISCUSSION

In the present study two well characterized murine models of liver injury have been combined in order to elucidate the interplay of cholestasis and HBsAg induced tumorigenesis [11, 18, 22, 23]. The extent of liver damage, cholestasis, and fibrosis was enhanced in Abcb4^−/−^/HBsAg^+/−^ mice. Activation of proto-oncogene c-Jun, transcription factor STAT3, and tumorigenesis were even potentiated by simultaneous damage. This is the first time that a ‘second hit’ mouse model for genetically determined cholestasis and HBsAg-induced liver damage generating carcinogenesis has been described.

Remarkably, the current study demonstrates aggravation of liver disease by intracellular HBsAg accumulation without viral infection in case of simultaneous labefaction. Primary biliary fibrosis and biliary atresia with additional HBV infection has been described in clinical studies [24, 25]. In addition, biliary diseases might even be attributed or caused by HBV infection [26, 27]. Especially, patients with immunosuppression and chronic HBV infection are at high risk of developing fibrosing cholestatic hepatitis, an aggressive and mostly fatal form of viral hepatitis [14]. Our data strengthen the idea that intracellular HBsAg accumulation might contribute to the aggravation of HBV associated cholestasis.

In 1998, a so called ‘two hit’ theory proposed steatosis as first hit sensitizing liver tissue to a variety of second hits like oxidative stress leading to necroinflammation and fibrosis [28]. With regard to our current results, HBsAg induced storage disease might be considered as a ‘second-hit’ providing the potential to enforce carcinogenesis in simultaneous chronic liver damage. This hypothesis is strengthened by the fact that patients with high HBV surface antigen levels develop HCC dose dependently [16, 17].

Increased ALT activity in Abcb4^−/−^/HBsAg^+/−^ mice nearly reflects the sum of individual ALT levels in corresponding transgenic/knock out controls and thus suggests an additive effect of both liver diseases. Liver cell damage, caused by a lack of Abcb4-transporters is primarily localized to the periportal area due to regurgitation of bile acids from leaky bile ducts whereas in HBsAg^+/−^ mice liver cell damage is homogenously distributed throughout the whole lobules. From the histopathological point of view cumulation of these damages might be possible. These findings correlate with enhanced cholangitis/pericholangitis and bile duct proliferation. Interestingly, serum bile salt concentrations correlate with serum ALT. It has been shown recently that serum bile acid concentrations >10 μM caused hepatocellular damage reflected by enhanced ALT levels [29]. Furthermore, BAs may behave as cancer promoters through indirect mechanisms involving oxidative stress and DNA damage, as well as they can act as selection agents for apoptosis-resistant cells [30]. On the other hand, tauroursodeoxycholic acid (TUDCA) protects hepatocytes from bile acid-induced apoptosis via activation of survival pathways [31]. Another study demonstrated that unconjugated UDCA provides protection against the cytotoxicity due to toxic BAs, the stimulation of hepatobiliary secretion, antioxidant activity, due in part to an enhancement in glutathione levels, and the inhibition of liver cell apoptosis [30]. It has been demonstrated that bile acids can induce endoplasmic reticulum stress, which in turn stimulates apoptosis, in a hydrophobicity dependent manner [32]. On the other hand it has been suggested that ER-stress can alter bile acid metabolism [33]. The absolute amount of TUDCA is unchanged but the relative amount of TUDCA decreased by the total increase of BAs (Supplementary Table 1). Nevertheless, the absolute increase of BAs might be a cumulative effect of enhanced BA-concentrations in both models (Figure 2) and of ER-stress-altered BA-metabolism [33]. According to these observations, reduced TUDCA levels in Abcb4^−/−^/HBsAg^+/−^ mice presumably contributed to deleterious effects of enhanced serum bile acid concentrations. The current data suggest that the combination of the underlying biliary components of individual damage might critically influence the exacerbation of pathologic effects in Abcb4^−/−^/HBsAg^+/−^ mice.

Beyond the finding, that a combination of murine models caused a cumulative effect on hepatocellular damage and fibrosis, development of hepatic tumors was potentiated in Abcb4^−/−^/HBsAg^+/−^ mice. In contrast to human beings, where HCC development is mostly based on hepatic cirrhosis, Abcb4^−/−^/HBsAg^+/−^ mice displayed a high tumor development without underlying cirrhosis. In HBV infection, the risk of HCC development is primary linked to cirrhosis, as HCC incidence rises from 0.6 per 100 persons in chronic HBV infected patients without cirrhosis to 3.7 in HBV cirrhosis [17, 34]. JNK, c-JUN, and STAT-3 activation were pronounced in Abcb4^−/−^/HBsAg^+/−^ mice (Figure 4C). JNK- as well as downstream c-Jun-, and STAT-3-activation mediate several cellular processes, including proliferation, and thus are critical for HCC induction [35, 36]. It might be speculated that a simultaneous activation of these tumorigenic pathways is responsible for aggravated carcinogenesis in Abcb4^−/−^/HBsAg^+/−^ mice.

Interestingly, reduced HBsAg transgene expression barely had any effect on UPR and UPR induced apoptosis in Abcb4^−/−^/HBsAg^+/−^ mice (compared to HBsAg controls). Reduced HBsAg accumulation and unchanged UPR induction is different to our previous observations, where loss of HBsAg expression by DNA methylation ameliorated cell stress and liver integrity in HBsAg^+/−^ mice [37]. Animals with reduced HBsAg expression due to DNA methylation were excluded from the experiment in the current study. Furthermore, ER stress and UPR might play a role in Abcb4^−/−^ mice, although CHOP and thereby ER stress dependent apoptosis were not induced (Figure 1C). Indeed, it has been described before that intrahepatic cholestasis due to accumulation of bile acids in the liver was associated with ER stress [38]. However, the cellular pathways that regulate the hepatic UPR and ER stress in cholestasis remain undefined [39]. The unfolded protein response (UPR) is generally activated in solid tumors and results in tumor cell anti-apoptosis and drug resistance [40]. On the other hand, it has been demonstrated that induction of ER-stress is essential for hepatocarcinogenesis [41]. A mouse model describing bile duct ligation combined with alpha-1 antitrypsin mutation PiZZ causing an ER storage disease has been published previously [42]. The authors concluded, that patients with α1-AT deficiency might be more susceptible to the development of severe liver disease when suffering from concomitant cholestatic liver injury and vice versa [42]. Our study confirms the conclusion that ER storage disease exacerbates cholestasis.

A limiting factor of our study might be a slight change of tumor incidence in the murine Abcb4^−/−^ model during the last years. In a formerly published study we described 100% incidence of hepatic tumors in Abcb4^−/−^ mice aged 52 weeks [43]. Hygienic housing conditions and analysis for the presence of mouse pathogens as well as subsequent therapy have been improved several times ever since in the animal facility. Influences of antibiotics regarding liver fibrosis and alterations of gut microbiota to hepatic fibrogenesis are well known [44, 45]. Thus reduction in HCC incidence over the years in Abcb4^−/−^ mice are most likely caused by improvements of hygienic status [44, 45].

In summary we conclude that hepatic injury, caused by cholestasis and HBsAg induced storage disease, reflects a model of simultaneous liver damage with enhanced carcinogenesis. Our data suggest that concurrent biliary liver disease provides a ‘second-hit’ which enforces HBsAg-associated carcinogenesis by the potentiated activation of proto-oncogene cJun and STAT3.

## MATERIALS AND METHODS

### Animal experimentation

The present study was performed with permission of the State of Hesse, Regierungspraesidium Giessen, according to Section 8 of the German Law for the Protection of Animals and conforms to the NIH Guide for the Care and Use of Laboratory Animals. Transgenic mice were maintained at the Central Animal Laboratory of the Justus-Liebig-University Giessen under specified pathogen-free conditions. All experiments were approved by the Committee on the ethics of Animal Experiments of the Regierungspraesidium Giessen, Giessen, Germany (permit number: V54-19c 2015c GI20/10 Nr. A36/2011, Nr. A5/2012 and Nr. 52_2011).

BALB/c-Abcb4 mice (C.FVB(129P2)-Abcb4^tm1Bor^ herein called Abcb4^−/−^ mice) were bred and housed as described previously [22]. Characterization of Abcb4^−/−^ genotype, sample collection, and routine analysis have been described elsewhere [22]. Generation and characteristics of transgenic lineages Tg(Alb-1HBV) (C57BL/6J-Tg (Alb1HBV) 44Bri/J) have been described [11]. These mice were backcrossed to BALB/cJ background (C.B6J-Tg (Alb1HBV) 44Bri N10 herein called HBsAg^+/−^ mice) for 9 generations. HBsAg^+/−^ mice were crossed with Abcb4^−/−^ mice, resulting in the F2 generation BALB/c-Abcb4/Alb1-HBV hybrid mice (C.Cg-Tg (Alb1HBV) 44Bri-Abcb4^tm1Bor^ herein called Abcb4^−/−^/HBsAg^+/−^ mice).

Mice were sacrificed at ages of 7-8 (young), 12-16 (middle), and 52 weeks (old) (n=4-5 per age and sex). Livers were collected and underwent morphological diagnosis. Remaining samples were preserved for analyses as indicated. Serum samples were stored at −80°C until analysis of alanine aminotransferases (ALT), as well as alkaline phosphatase (AP) by routine clinical chemistry on a Reflotron Plus Analyzer (Roche, Mannheim, Germany).

### Histology

Histology and immunohistochemistry were performed as described before [46]. Quantification of collagenous tissue was performed by assessment of stained area using ImageJ-software [11].

### Immunohistochemistry

Immunohistochemistry was performed on paraffin sections (5 μm) by using ImmPRESS Peroxidase Detection Reagents (Vector Laboratories) and antibodies specific for HBsAg (monoclonal mouse antibody MA18/7) [47], Ki-67 (RM-9106-SO, Thermo Scientific, Dreieich, Germany), Typ IV collagen (10760, Progen, Heidelberg, Germany), glutamine synthetase (GTX 109121, GeneTex, Irvine, USA). The colour reaction was carried out with VECTOR VIP Peroxidase Substrate Kit or DAB Peroxidase Substrate Kit, (Vector Laboratories).

### Western blotting

Sample preparation, sodium dodecyl sulfate-polyacrylamide gel electrophoresis and Western blotting was performed as described before [48]. Total liver lysates were analyzed by using antibodies against HBsAg (20-HR20, Fitzgerald, Acton, Ma, USA), phospho-PERK (16F8, Cell Signaling, Beverly, MA, USA), phospho-eIF2α (119A11, Cell Signaling), β-actin (13E5, Cell Signaling), c-Jun (60A8, Cell Signaling), phospho-c-Jun (D47G9, Cell Signaling), phospho-SAPK/JNK (81E11, Cell Signaling), STAT3 (79D7, Cell Signaling), phospho-STAT3 (D3A7, Cell Signaling).

### Bile acid analysis

Bile acids in serum were analyzed by UHPLC-MS/MS essentially as described before [49].

### Statistical analysis

Statistical analysis was performed with SPSS V.17.0 (SPSS Inc.) and GraphPad Prism5 (GraphPas Software Inc). For non-normally distributed parameters, Mann-Whitney U test and Spearman rank test were applied. Bivariate correlation of ALT and BA was performed with two tailed Spearman test. Enhanced tumor incidence was calculated with Chi^2^ statistics. The results are presented as mean ±SEM. *p<0.05 was considered significant.

## SUPPLEMENTARY FIGURE AND TABLE





## References

[1] Jacquemin E (2012). Progressive familial intrahepatic cholestasis. Clin Res Hepatol Gastroenterol.

[2] Linton KJ (2015). Lipid flopping in the liver. Biochem Soc Trans.

[3] Fickert P, Fuchsbichler A, Wagner M, Zollner G, Kaser A, Tilg H, Krause R, Lammert F, Langner C, Zatloukal K, Marschall HU, Denk H, Trauner M (2004). Regurgitation of bile acids from leaky bile ducts causes sclerosing cholangitis in Mdr2 (Abcb4) knockout mice. Gastroenterology.

[4] Chisari FV, Isogawa M, Wieland SF (2010). Pathogenesis of hepatitis B virus infection. Pathol Biol (Paris).

[5] Schuch A, Hoh A, Thimme R (2014). The role of natural killer cells and CD8(+) T cells in hepatitis B virus infection. Front Immunol.

[6] Meuleman P, Libbrecht L, Wieland S, De VR, Habib N, Kramvis A, Roskams T, Leroux-Roels G (2006). Immune suppression uncovers endogenous cytopathic effects of the hepatitis B virus. J Virol.

[7] Pol S (2013). Management of HBV in immunocompromised patients. Liver Int.

[8] Shouval D, Shibolet O (2013). Immunosuppression and HBV reactivation. Semin Liver Dis.

[9] Chisari FV, Filippi P, McLachlan A, Milich DR, Riggs M, Lee S, Palmiter RD, Pinkert CA, Brinster RL (1986). Expression of hepatitis B virus large envelope polypeptide inhibits hepatitis B surface antigen secretion in transgenic mice. J Virol.

[10] Chisari FV, Klopchin K, Moriyama T, Pasquinelli C, Dunsford HA, Sell S, Pinkert CA, Brinster RL, Palmiter RD (1989). Molecular pathogenesis of hepatocellular carcinoma in hepatitis B virus transgenic mice. Cell.

[11] Churin Y, Roderfeld M, Stiefel J, Wurger T, Schroder D, Matono T, Mollenkopf HJ, Montalbano R, Pompaiah M, Reifenberg K, Zahner D, Ocker M, Gerlich W (2014). Pathological impact of hepatitis B virus surface proteins on the liver is associated with the host genetic background. PLoS One.

[12] Montalbano R, Honrath B, Wissniowski TT, Elxnat M, Roth S, Ocker M, Quint K, Churin Y, Roederfeld M, Schroeder D, Glebe D, Roeb E, Di Fazio P (2016). Exogenous hepatitis B virus envelope proteins induce endoplasmic reticulum stress: involvement of cannabinoid axis in liver cancer cells. Oncotarget.

[13] Wirth S, Guidotti LG, Ando K, Schlicht HJ, Chisari FV (1995). Breaking tolerance leads to autoantibody production but not autoimmune liver disease in hepatitis B virus envelope transgenic mice. J Immunol.

[14] Churin Y, Roderfeld M, Roeb E (2015). Hepatitis B virus large surface protein: function and fame. Hepatobiliary Surg Nutr.

[15] Reifenberg K, Hildt E, Lecher B, Wiese E, Nusser P, Ott S, Yamamura K, Rutter G, Lohler J (2006). IFNgamma expression inhibits LHBs storage disease and ground glass hepatocyte appearance, but exacerbates inflammation and apoptosis in HBV surface protein-accumulating transgenic livers1. Liver Int.

[16] Tseng TC, Liu CJ, Yang HC, Su TH, Wang CC, Chen CL, Hsu CA, Kuo SF, Liu CH, Chen PJ, Chen DS, Kao JH (2013). Serum hepatitis B surface antigen levels help predict disease progression in patients with low hepatitis B virus loads. Hepatology.

[17] Kawanaka M, Nishino K, Nakamura J, Oka T, Urata N, Goto D, Suehiro M, Kawamoto H, Kudo M, Yamada G (2014). Quantitative Levels of Hepatitis B Virus DNA and Surface Antigen and the Risk of Hepatocellular Carcinoma in Patients with Hepatitis B Receiving Long-Term Nucleos(t)ide Analogue Therapy. Liver Cancer.

[18] Trierweiler C, Hockenjos B, Zatloukal K, Thimme R, Blum HE, Wagner EF, Hasselblatt P (2016). The transcription factor c-JUN/AP-1 promotes HBV-related liver tumorigenesis in mice. Cell Death Differ.

[19] Malhi H, Kaufman RJ (2011). Endoplasmic reticulum stress in liver disease1. J Hepatol.

[20] Jörs S, Jeliazkova P, Ringelhan M, Thalhammer J, Durl S, Ferrer J, Sander M, Heikenwalder M, Schmid RM, Siveke JT, Geisler F (2015). Lineage fate of ductular reactions in liver injury and carcinogenesis. J Clin Invest.

[21] Tarlow BD, Pelz C, Naugler WE, Wakefield L, Wilson EM, Finegold MJ, Grompe M (2014). Bipotential adult liver progenitors are derived from chronically injured mature hepatocytes. Cell Stem Cell.

[22] Roderfeld M, Rath T, Voswinckel R, Dierkes C, Dietrich H, Zahner D, Graf J, Roeb E (2010). Bone marrow transplantation demonstrates medullar origin of CD34+ fibrocytes and ameliorates hepatic fibrosis in Abcb4−/− mice. Hepatology.

[23] Zhou M, Learned RM, Rossi SJ, DePaoli AM, Tian H, Ling L (2016). Engineered fibroblast growth factor 19 reduces liver injury and resolves sclerosing cholangitis in Mdr2-deficient mice. Hepatology.

[24] Rigopoulou EI, Zachou K, Gatselis NK, Papadamou G, Koukoulis GK, Dalekos GN (2013). Primary biliary cirrhosis in HBV and HCV patients: Clinical characteristics and outcome. World J Hepatol.

[25] Yaghobi R, Didari M, Gramizadeh B, Rahsaz M, Heidari T, Banihashemi M, Kargar M (2011). Study of viral infections in infants with biliary atresia. Indian J Pediatr.

[26] Burgart LJ (1998). Cholangitis in viral disease. Mayo Clin Proc.

[27] Gupta E, Chakravarti A (2008). Viral infections of the biliary tract. Saudi J Gastroenterol.

[28] Day CP, James OF (1998). Steatohepatitis: a tale of two “hits”?. Gastroenterology.

[29] Song P, Zhang Y, Klaassen CD (2011). Dose-response of five bile acids on serum and liver bile Acid concentrations and hepatotoxicty in mice. Toxicol Sci.

[30] Perez MJ, Briz O (2009). Bile-acid-induced cell injury and protection. World J Gastroenterol.

[31] Schoemaker MH, Conde de la RL, Buist-Homan M, Vrenken TE, Havinga R, Poelstra K, Haisma HJ, Jansen PL, Moshage H (2004). Tauroursodeoxycholic acid protects rat hepatocytes from bile acid-induced apoptosis via activation of survival pathways. Hepatology.

[32] Adachi T, Kaminaga T, Yasuda H, Kamiya T, Hara H (2014). The involvement of endoplasmic reticulum stress in bile acid-induced hepatocellular injury1. J Clin Biochem Nutr.

[33] Flowers MT, Keller MP, Choi Y, Lan H, Kendziorski C, Ntambi JM, Attie AD (2008). Liver gene expression analysis reveals endoplasmic reticulum stress and metabolic dysfunction in SCD1-deficient mice fed a very low-fat diet1. Physiol Genomics.

[34] Fattovich G, Bortolotti F, Donato F (2008). Natural history of chronic hepatitis B: special emphasis on disease progression and prognostic factors. J Hepatol.

[35] Eferl R, Ricci R, Kenner L, Zenz R, David JP, Rath M, Wagner EF (2003). Liver tumor development. c-Jun antagonizes the proapoptotic activity of p53. Cell.

[36] He G, Karin M (2011). NF-kappaB and STAT3 - key players in liver inflammationand cancer. Cell Res.

[37] Graumann F, Churin Y, Tschuschner A, Reifenberg K, Glebe D, Roderfeld M, Roeb E (2015). Genomic Methylation Inhibits Expression of Hepatitis B Virus Envelope Protein in Transgenic Mice: A Non-Infectious Mouse Model to Study Silencing of HBV Surface Antigen Genes. PLoS One.

[38] Bochkis IM, Rubins NE, White P, Furth EE, Friedman JR, Kaestner KH (2008). Hepatocyte-specific ablation of Foxa2 alters bile acid homeostasis and results in endoplasmic reticulum stress1. Nat Med.

[39] Dai BH, Geng L, Wang Y, Sui CJ, Xie F, Shen RX, Shen WF, Yang JM (2013). microRNA-199a-5p protects hepatocytes from bile acid-induced sustained endoplasmic reticulum stress1. Cell Death Dis.

[40] Tang J, Guo YS, Zhang Y, Yu XL, Li L, Huang W, Li Y, Chen B, Jiang JL, Chen ZN (2012). CD147 induces UPR to inhibit apoptosis and chemosensitivity by increasing the transcription of Bip in hepatocellular carcinoma1. Cell Death Differ.

[41] Shuda M, Kondoh N, Imazeki N, Tanaka K, Okada T, Mori K, Hada A, Arai M, Wakatsuki T, Matsubara O, Yamamoto N, Yamamoto M (2003). Activation of the ATF6, XBP1 and grp78 genes in human hepatocellular carcinoma: a possible involvement of the ER stress pathway in hepatocarcinogenesis1. J Hepatol.

[42] Mencin A, Seki E, Osawa Y, Kodama Y, De MS, Knowles M, Brenner DA (2007). Alpha-1 antitrypsin Z protein (PiZ) increases hepatic fibrosis in a murine model of cholestasis. Hepatology.

[43] Roderfeld M, Rath T, Lammert F, Dierkes C, Graf J, Roeb E (2010). Innovative immunohistochemistry identifies MMP-9 expressing macrophages at the invasive front of murine HCC. World J Hepatol.

[44] Seki E, De MS, Osterreicher CH, Kluwe J, Osawa Y, Brenner DA, Schwabe RF (2007). TLR4 enhances TGF-beta signaling and hepatic fibrosis. Nat Med.

[45] Xie G, Wang X, Liu P, Wei R, Chen W, Rajani C, Hernandez BY, Alegado R, Dong B, Li D, Jia W (2016). Distinctly altered gut microbiota in the progression of liver disease. Oncotarget.

[46] Roderfeld M, Rath T, Pasupuleti S, Zimmermann M, Neumann C, Churin Y, Dierkes C, Voswinckel R, Barth PJ, Zahner D, Graf J, Roeb E (2012). Bone marrow transplantation improves hepatic fibrosis in Abcb4−/− mice via Th1 response and matrix metalloproteinase activity. Gut.

[47] Heermann KH, Goldmann U, Schwartz W, Seyffarth T, Baumgarten H, Gerlich WH (1984). Large surface proteins of hepatitis B virus containing the pre-s sequence. J Virol.

[48] Henkel C, Roderfeld M, Weiskirchen R, Berres ML, Hillebrandt S, Lammert F, Meyer HE, Stuhler K, Graf J, Roeb E (2006). Changes of the hepatic proteome in murine models for toxically induced fibrogenesis and sclerosing cholangitis. Proteomics.

[49] Sawitza I, Kordes C, Gotze S, Herebian D, Haussinger D (2015). Bile acids induce hepatic differentiation of mesenchymal stem cells. Sci Rep.

