# Supplementary material for: Hepatitis B virus surface proteins accelerate cholestatic injury and tumor progression in Abcb4-knockout mice
Source: Oncotarget. 2017 Feb 2;8(32):52560–70. doi: 10.18632/oncotarget.15003 (PMC5581050; doi:10.18632/oncotarget.15003)
Supplement: Supplementary file 1 [file oncotarget-08-52560-s001.pdf]

# Hepatitis B virus surface proteins accelerate cholestatic injury and tumor progression in Abcb4-knockout mice

## SUPPLEMENTARY FIGURE AND TABLE

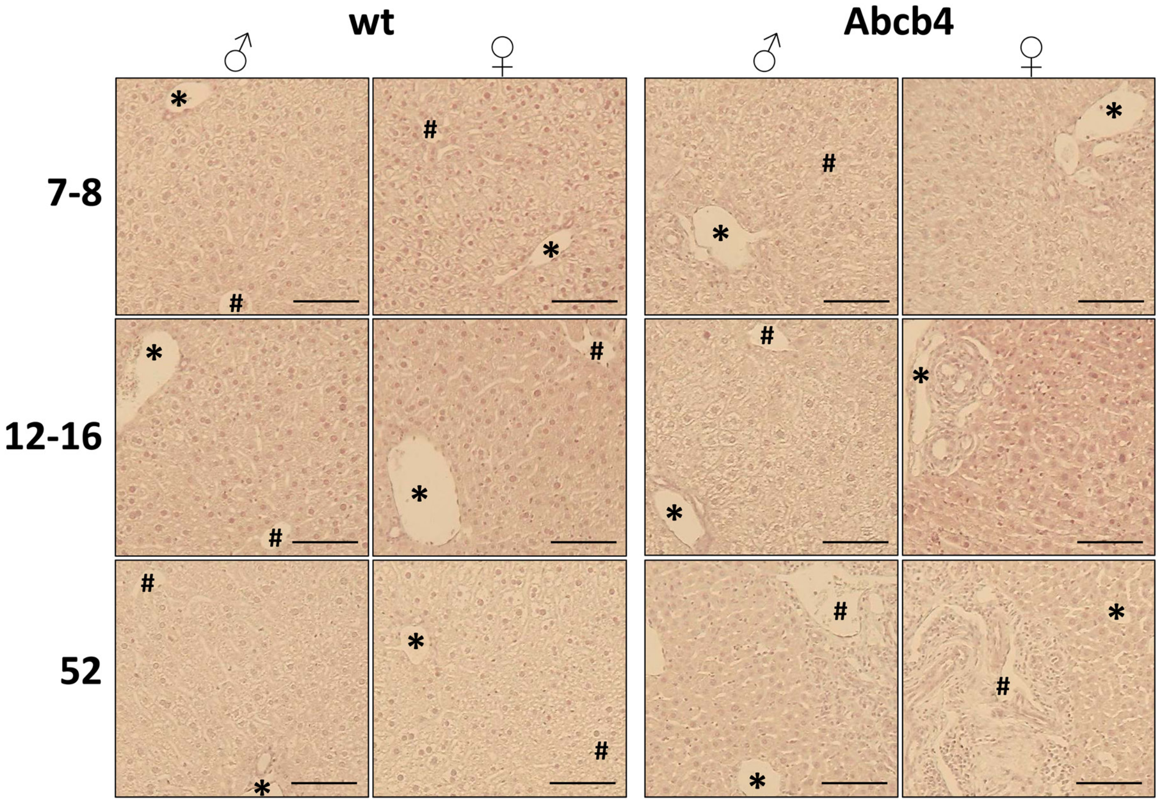


**Supplementary Figure 1: Negative controls for the staining of HBsAg in wild type mice and Abcb4$^{-/-}$ mice.** Immunohistochemistry for HBsAg expression in wild type- and Abcb4$^{-/-}$ mice. # central vein, * portal field. Magnification x100, scale bars 100 µm. Representative micrographs are shown.

**Supplementary Table 1: Serum bile acid concentrations (μM, mean ± standard deviation).** Cholic acid (CA), chenodeoxycholic acid (CDCA), deoxycholic acid (DCA), murideoxycholic acid (MDCA), ursodeoxycholic acid (UDCA), hyodeoxycholic acid (HDCA), muricholic acids (α MCA, β MCA, ω MCA), glycocholic acid (GCA), glycochenodeoxycholic acid (GCDCA), glycodeoxycholic acid (GDCA), glycoursodeoxycholic acid (GUDCA), glycohyodeoxycholic acid (GHDCA), taurocholic acid (TCA), taurohyocholic acid (THCA), taurochenodeoxycholic acid (TCDCA), taurodeoxycholic acid (TDCA), taurolithocholic acid (TLCA), tauroursodeoxycholic acid (TUDCA), tauromurideoxycholic acid (TMDCA), tauromuricholic acids (α MCA, β MCA, ω MCA), and taurohyodeoxycholic acid (THDCA).

See Supplementary File 1
