# Supplementary material for: Hepatitis B virus surface proteins accelerate cholestatic injury and tumor progression in Abcb4-knockout mice
Source: Oncotarget. 2017 Feb 2;8(32):52560–70. doi: 10.18632/oncotarget.15003 (PMC5581050; doi:10.18632/oncotarget.15003)
Supplement: Supplementary file 2 [file oncotarget-08-52560-s002.docx]

|  |  | **Taurine-conjugates** | | | | | | | | | | | **Glycine-conjugates** | | | | | **unconjugated** | | | | | | | | |
| --- | --- | --- | --- | --- | --- | --- | --- | --- | --- | --- | --- | --- | --- | --- | --- | --- | --- | --- | --- | --- | --- | --- | --- | --- | --- | --- |
| **age /weeks** | **group** | **T-ω-MCA** | **T-α-MCA** | **T-β-MCA** | **THCA** | **TCA** | **TMDCA** | **TUDCA** | **THDCA** | **TCDCA** | **TDCA** | **TLCA** | **GCA** | **GUDCA** | **GHDCA** | **GCDCA** | **GDCA** | **ω-MCA** | **α-MCA** | **β-MCA** | **CA** | **MDCA** | **UDCA** | **HDCA** | **CDCA** | **DCA** |
| **8** | **wt** | 0.052 ±0.036 | 0.002 ±0.003 | 0.031 ±0.014 | 0.008 ±0.002 | 0.64 ±0.24 | 0.005 ±0.004 | 0.008 ±0.003 | 0.027 ±0.01 | 0.028 ±0.013 | 0.15 ±0.093 | 0.27±0.25 | 0.033 ±0.077 | 0.008 ±0.003 | 0.005 ±0.002 | 0.011 ±0.006 | 0.02 ±0.01 | 0.14 ±0.06 | 0.025 ±0.014 | 0.12 ±0.029 | 0.21 ±0.21 | 0.016 ±0.011 | 0.01 ±0.004 | 0.009 ±0.004 | 0.025 ±0.014 | 0.12 ±0.031 |
|  | **Abcb4** | 1.4 ±1.3 | 0.022 ±0.016 | 1.3 ±1.6 | 0.011 ±0.007 | 13.3 ±13.3 | 0.12 ±0.12 | 0.11 ±0.11 | 0.21 ±0.16 | 0.16 ±0.12 | 0.95 ±0.82 | 0.031 ±0.019 | 0.044 ±0.02 | 0.005 ±0.003 | 0.005 ±0.002 | 0.004 ±0.003 | 0.018 ±0.005 | 0.32 ±0.25 | 0.024 ±0.008 | 0.29 ±0.3 | 0.1 ±0.043 | 0.032 ±0.031 | 0.012 ±0.004 | 0.017 ±0.01 | 0.008 ±0.002 | 0.17 ±0.067 |
|  | **HBsAg** | 0.19 ±0.043 | 0.007 ±0.002 | 0.16 ±0.073 | 0.008 ±0.003 | 1.8 ±1.1 | 0.021 ±0.005 | 0.019 ±0.009 | 0.06 ±0.013 | 0.061 ±0.025 | 0.19 ±0.14 | 0.004 ±0.001 | 0.028 ±0.005 | 0.007 ±0.002 | 0.003 ±0.001 | 0.003 ±0.002 | 0.014 ±0.003 | 0.074 ±0.056 | 0.025 ±0.005 | 0.12 ±0.021 | 0.14 ±0.19 | 0.012 ±0.007 | 0.009 ±0.003 | 0.007 ±0.006 | 0.006 ±0.004 | 0.066 ±0.035 |
|  | **Abcb4/ HBsAg** | 1.5 ±0.69 | 0.026 ±0.019 | 1.3 ±0.98 | 0.009 ±0.004 | 10.4 ±8.0 | 0.088 ±0.035 | 0.078 ±0.049 | 0.16 ±0.078 | 0.19 ±0.1 | 0.55 ±0.36 | 0.010 ±0.004 | 0.039 ±0.014 | 0.006 ±0.003 | 0.003 ±0.001 | 0.004 ±0.002 | 0.014 ±0.006 | 0.22 ±0.065 | 0.022 ±0.007 | 0.18 ±0.035 | 0.13 ±0.15 | 0.022 ±0.01 | 0.011 ±0.003 | 0.01 ±0.003 | 0.007 ±0.004 | 0.19 ±0.12 |
| **12-16** | **wt** | 0.15 ±0.15 | 0.02 ±0.029 | 0.12 ±0.14 | 0.009 ±0.003 | 1.4 ±1.4 | 0.039 ±0.041 | 0.025 ±0.023 | 0.05 ±0.029 | 0.055 ±0.057 | 0.26 ±0.19 | 0.004 ±0.001 | 0.023 ±0.006 | 0.008 ±0.003 | 0.003 ±0.002 | 0.002 ±0.002 | 0.012 ±0.004 | 0.23 ±0.12 | 0.037 ±0.012 | 0.15 ±0.072 | 0.17 ±0.17 | 0.013 ±0.006 | 0.011 ±0.004 | 0.011 ±0.005 | 0.013 ±0.014 | 0.12 ±0.076 |
|  | **Abcb4** | 1.3 ±1.6 | 0.015 ±0.019 | 1.0 ±1.6 | 0.011 ±0.010 | 10.5 ±13.0 | 0.13 ±0.2 | 0.1 ±0.13 | 0.22 ±0.29 | 0.14 ±0.14 | 0.77 ±0.97 | 0.007 ±0.003 | 0.045 ±0.024 | 0.006 ±0.003 | 0.004 ±0.001 | 0.005 ±0.002 | 0.013 ±0.004 | 0.28 ±0.21 | 0.027 ±0.006 | 0.17 ±0.062 | 0.073 ±0.027 | 0.014 ±0.011 | 0.01 ±0.008 | 0.012 ±0.007 | 0.004 ±0.003 | 0.27 ±0.23 |
|  | **HBsAg** | 0.65 ±0.38 | 0.013 ±0.01 | 0.87 ±0.66 | 0.009 ±0.004 | 2.5 ±0.80 | 0.052 ±0.03 | 0.031 ±0.015 | 0.088 ±0.05 | 0.12 ±0.058 | 0.13 ±0.13 | 0.007 ±0.002 | 0.032 ±0.01 | 0.010 ±0.003 | 0.004 ±0.002 | 0.003 ±0.002 | 0.018 ±0.005 | 0.22 ±0.16 | 0.024 ±0.006 | 0.16 ±0.059 | 0.083 ±0.055 | 0.019 ±0.013 | 0.012 ±0.01 | 0.018 ±0.025 | 0.019 ±0.018 | 0.088 ±0.045 |
|  | **Abcb4/ HBsAg** | 4.7 ±4.7 | 0.22 ±0.56 | 5.4 ±4.6 | 0.015 ±0.013 | 18.0 ±11.4 | 0.2 ±0.13 | 0.11 ±0.088 | 0.37 ±0.30 | 0.75 ±0.74 | 0.55 ±0.30 | 0.031 ±0.02 | 0.05 ±0.022 | 0.01 ±0.003 | 0.004 ±0.001 | 0.005 ±0.002 | 0.02 ±0.005 | 0.52 ±0.31 | 0.022 ±0.005 | 0.34 ±0.22 | 0.21 ±0.31 | 0.021 ±0.007 | 0.011 ±0.005 | 0.021 ±0.009 | 0.010 ±0.008 | 0.19 ±0.081 |
| **52** | **wt** | 0.16 ±0.084 | 0.005 ±0.003 | 0.13 ±0.052 | 0.010 ±0.002 | 1.5 ±0.70 | 0.011 ±0.006 | 0.016 ±0.005 | 0.039 ±0.017 | 0.034 ±0.008 | 0.13 ±0.06 | 0.005 ±0 | 0.031 ±0.006 | 0.009 ±0.003 | 0.003 ±0.001 | 0.004 ±0.002 | 0.013 ±0.005 | 0.18 ±0.096 | 0.021 ±0.008 | 0.10 ±0.012 | 0.046 ±0.004 | 0.020 ±0.014 | 0.008 ±0.003 | 0.006 ±0.003 | 0.020 ±0.015 | 0.12 ±0.035 |
|  | **Abcb4** | 10.1 ±8.7 | 0.14 ±0.12 | 13.0 ±10.1 | 0.020 ±0.011 | 57.0 ±41.9 | 0.86 ±0.79 | 0.77 ±0.69 | 1.3 ±1.4 | 1.5 ±1.1 | 5.5 ±4.7 | 0.017 ±0.009 | 0.081 ±0.038 | 0.010 ±0.002 | 0.004 ±0.001 | 0.004 ±0.002 | 0.018 ±0.003 | 1.6 ±1.3 | 0.03 ±0.007 | 0.45 ±0.28 | 0.22 ±0.24 | 0.019 ±0.017 | 0.04 ±0.052 | 0.027 ±0.039 | 0.018 ±0.013 | 1.4 ±1.1 |
|  | **HBsAg** | 1.1 ±0.75 | 0.046 ±0.041 | 1.7 ±1.4 | 0.009 ±0.003 | 6.1 ±3.6 | 0.071 ±0.05 | 0.064 ±0.042 | 0.11 ±0.072 | 0.23 ±0.13 | 0.32 ±0.16 | 0.008 ±0.002 | 0.034 ±0.008 | 0.009 ±0.003 | 0.004 ±0.001 | 0.004 ±0.001 | 0.017 ±0.003 | 0.34 ±0.22 | 0.023 ±0.007 | 0.21 ±0.13 | 0.086 ±0.08 | 0.005 ±0.009 | 0.012 ±0.006 | 0.012 ±0.005 | 0.015 ±0.011 | 0.19 ±0.081 |
|  | **Abcb4/ HBsAg** | 16.5 ±7.6 | 0.23 ±0.15 | 26.1 ±12.3 | 0.13 ±0.1 | 92.6 ±44.9 | 0.50 ±0.26 | 0.58 ±0.38 | 0.64 ±0.34 | 3.1 ±1.5 | 3.6 ±2.3 | 0.017 ±0.006 | 0.11 ±0.057 | 0.008 ±0.003 | 0.004 ±0.002 | 0.005 ±0.003 | 0.018 ±0.002 | 1.6 ±1.4 | 0.024 ±0.005 | 0.75 ±0.39 | 0.30 ±0.28 | 0.020 ±0.014 | 0.023 ±0.027 | 0.032 ±0.018 | 0.017 ±0.01 | 0.75 ±0.40 |

Suppl. table1: Serum bile acid concentrations (µM, mean ± standard deviation). Cholic acid (CA), chenodeoxycholic acid (CDCA), deoxycholic acid (DCA), murideoxycholic acid (MDCA), ursodeoxycholic acid (UDCA), hyodeoxycholic acid(HDCA), muricholic acids (α MCA, β MCA, ω MCA), glycocholic acid (GCA),

glycochenodeoxycholic acid (GCDCA), glycodeoxycholic acid (GDCA), glycoursodeoxycholic acid (GUDCA), glycohyodeoxycholic acid (GHDCA), taurocholic acid

(TCA), taurohyocholic acid (THCA), taurochenodeoxycholic acid (TCDCA), taurodeoxycholic acid (TDCA), taurolithocholic acid (TLCA), tauroursodeoxycholic acid (TUDCA), tauromurideoxycholic acid (TMDCA), tauromuricholic acids (α MCA, β MCA, ω MCA), and taurohyodeoxycholic acid (THDCA).
